# A Quantitative Method for Assessment of Prescribing Patterns Using Electronic Health Records

**DOI:** 10.1371/journal.pone.0075214

**Published:** 2013-10-10

**Authors:** Dukyong Yoon, Inwhee Park, Martijn J. Schuemie, Man Young Park, Ju Han Kim, Rae Woong Park

**Affiliations:** 1 Department of Biomedical Informatics, Ajou University School of Medicine, Suwon, Korea; 2 Department of Nephrology, Ajou University School of Medicine, Suwon, Korea; 3 Janssen Research and Development LLC, Titusville, New Jersey, United States of America; 4 Seoul National University Biomedical Informatics, Seoul National University College of Medicine, Seoul, Korea; 5 Center for Clinical Epidemiology and Biostatistics, and Department of Biostatistics and Epidemiology, Perelman School of Medicine at the University of Pennsylvania, Philadelphia, Pennsylvania, United States of America; 6 Center for Pharmacoepidemiololgy Research and Training, Perelman School of Medicine at the University of Pennsylvania, Philadelphia, Pennsylvania, United States of America; University of Vermont, United States of America

## Abstract

**Background:**

Most available quality indicators for hospitals are represented by simple ratios or proportions, and are limited to specific events. A generalized method that can be applied to diverse clinical events has not been developed. The aim of this study was to develop a simple method of evaluating physicians' prescription patterns for diverse events and their level of awareness of clinical practice guidelines.

**Methods and Findings:**

We developed a quantitative method called Prescription pattern Around Clinical Event (PACE), which is applicable to electronic health records (EHRs). Three discrete prescription patterns (intervention, maintenance, and discontinuation) were determined based on the prescription change index (PCI), which was calculated by means of the increase or decrease in the prescription rate after a clinical event. Hyperkalemia and Clostridium difficile-associated diarrhea (CDAD) were used as example cases. We calculated the PCIs of 10 drugs related to hyperkalemia, categorized them into prescription patterns, and then compared the resulting prescription patterns with the known standards for hyperkalemia treatment. The hyperkalemia knowledge of physicians was estimated using a questionnaire and compared to the prescription pattern. Prescriptions for CDAD were also determined and compared to clinical knowledge. Clinical data of 1698, 348, and 1288 patients were collected from EHR data. The physicians prescribing behaviors for hyperkalemia and CDAD were concordant with the standard knowledge. Prescription patterns were well correlated with individual physicians' knowledge of hyperkalemia (κ = 0.714). Prescribing behaviors according to event severity or clinical condition were plotted as a simple summary graph.

**Conclusion:**

The algorithm successfully assessed the prescribing patterns from the EHR data. The prescription patterns were well correlated with physicians' knowledge. We expect that this algorithm will enable quantification of prescribers' adherence to clinical guidelines and be used to facilitate improved prescribing practices.

## Introduction

Treatment adherence to clinical practice guidelines is emphasized because these guidelines help physicians keep their practice current [1]. Recent articles report that guideline-concordant treatments improve clinical outcomes and reduce mortality [2], [3]. Many evidence-based clinical guidelines, to which clinicians are expected to adhere in daily practice, are extant [4]. However, large deviations from standard clinical knowledge have been observed in actual clinical practice [5]–[7].

Many quality indicators for hospitals are available, such as those provided by the Agency for Healthcare Research and Quality; however, many of these are simple ratios or proportions that consider quantitative clinical outcomes, such as rates of admission, adverse events, and mortality. A quality indicator applied to a specific outcome usually cannot be used to measure other unintended clinical events. Although a hospital requires many clinical indicators, the quality status of a hospital can be understood only in the context of a comparison or relative ranking among hospitals. No generalized method that can be applied to diverse clinical events or behaviors of physicians has been developed. Furthermore, existing methods cannot evaluate whether physicians recognize clinical practice guidelines.

Because electronic health records (EHRs) are being increasingly adopted worldwide, large quantities of EHR data are becoming available for clinical studies [8], [9]. One opportunity provided by these data is gaining of insight into the prescribing patterns of specific clinicians or groups thereof, which enables monitoring, improving the quality of practice, and reducing harm due to adverse events. However, tools that facilitate this approach are limited.

Recently, Schuemie proposed the Longitudinal Evaluation of Observational Profiles of Adverse events Related to Drugs (LEOPARD), a method that allows detection of protopathic bias in longitudinal observational data by comparing the accumulated number of prescriptions before and after an adverse drug event (ADE) [10]. More prescriptions after an ADE than before indicated protopathic bias or confounding by indication. We hypothesized that this method can be used to reveal the prescription intention of clinicians; more prescriptions after a clinical event may reflect an intention to treat, whereas fewer prescriptions may reflect an intention to discontinue treatment; and an equal or similar number of prescriptions may reflect an intention to maintain treatment or lack of knowledge of the event. To accomplish this, we modified the LEOPARD algorithm and termed the ratio of the accumulated number of prescriptions of a specific drug before and after a specific event the prescription change index (PCI). We distinguished the following three prescription patterns based on both this index and specific cut-off values: “intervention pattern”, “discontinuation pattern”, and “maintenance pattern”.

We performed three proof-of-concept studies using hyperkalemia and *Clostridium difficile*-associated diarrhea (CDAD) as examples of clinical events. For the first study, we calculated the PCIs of 10 drugs used for hyperkalemia and compared the resulting prescription patterns with the known standard treatment as a crude validation. The second study showed whether the prescription pattern was an accurate representation of the physician's knowledge of the clinical event. The third study investigated the possible generalizability of the proposed method using CDAD as an example clinical event.

## Methods

### 1. Ethics statement

The institutional review board of Ajou University Hospital approved this study. Informed consent was waived by the review board because the retrospective data were analyzed anonymously.

### 2. PCI and prescription patterns

**Figure 1:**
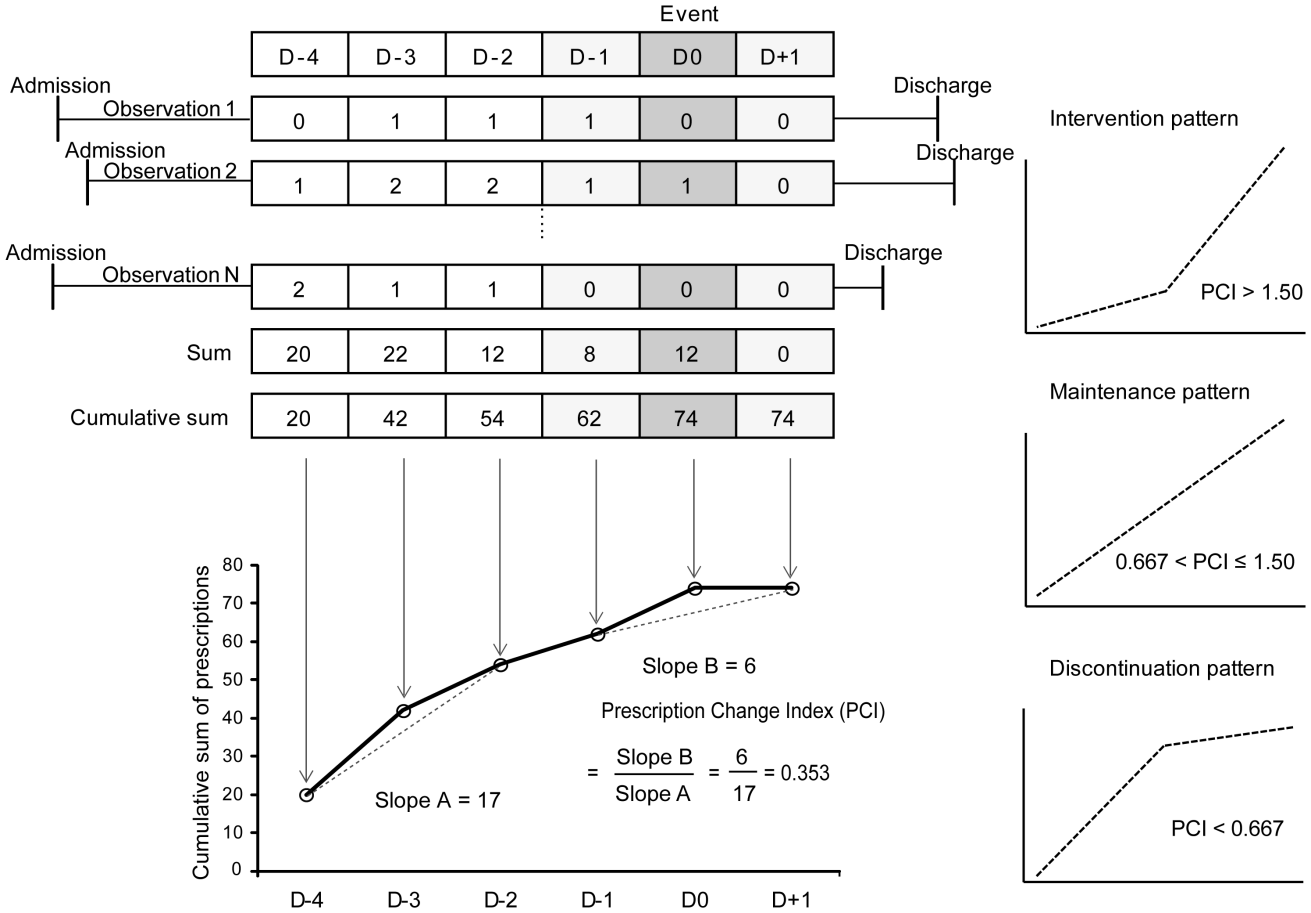
Overview of the prescription change index (PCI) and prescription pattern. The PCI reflects whether the number of prescriptions before and after a specific clinical event differed. The numerator represents the cumulative prescription count during the 2 days after an event occurred (D0, and D+1); the denominator represents the cumulative prescription count during the 2 days before an event occurred (D-3, and D-2). The PCI was categorized into the following three patterns: intervention, maintenance, and discontinuation. D-4, D-2, and D-1 represent 4, 2, and 1 day(s) before the event, respectively; D0 represents the day on which the event occurred; D+1 represents 1 day after the event.

To determine whether the number of prescriptions increased or decreased after a specific clinical event, we defined PCI as follows:
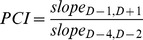
where, slope_t1,t2_ refers to the average slope of the cumulative sum of prescription counts from t1 to t2 (**Figure 1**). In the equation, D-4, D-2, and D-1 represent 4, 2, and 1 day(s) before the clinical events, respectively. D+1 represents 1 day after the clinical event. The numerator represents the cumulative count of prescriptions during the 2 days around event occurrence (D0, and D+1, where D0 represents the day on which the event occurred); the denominator represents the cumulative prescription count during the 2 days before the event occurred (D-3, and D-2). Thus, the equation reflects simply whether the number of drug prescriptions before and after a specific clinical event differed. For example, as seen in **Figure 1**, Slope A = (54-20)/2 = 17, Slope B = (74-62)/2 = 6, thus PCI = 6/17 = 0.353.

Three prescription patterns were defined as follows; the discontinuation pattern: reduced use of the drug which is thought to cause the event or which has a negative effect on the event; intervention pattern: increased use of the drug having positive treatment effects; maintenance pattern: continued use of the drug is independent of or proper in the event. A 1.5-fold reduction or increase in prescription number was set as the cut-off value; thus a PCI≤0.667 (1.000/1.500) was defined as a discontinuation pattern; >1.500 was defined as an intervention pattern; and others were classified as a maintenance pattern.

The observation period was from 4 days before to 1 day after the clinical event, whereas the observation period was confined to the duration of hospitalization. Thus, events that occurred at least 4 days after admission and 1 day before discharge were included in the analysis. If a patient experienced multiple clinical events during hospitalization, then each was counted separately. PCI for each drug was calculated from the observations, and the prescription pattern was determined based on the PCI.

### 3. Proof of concept

We extracted clinical data from the clinical data warehouse of Ajou University Hospital, which has been reported and described previously [5], [11]–[17]. The data warehouse contains 18 years (June 1994–June 2012) of clinical data including ∼105.6 million prescriptions, ∼141.6 million laboratory tests and ∼1.7 million admissions from ∼2.3 million patients.

#### 3.1 Hypothesis testing: application to hyperkalemia

Ten drugs (amlodipine, calcium gluconate, hydrochlorothiazide, ibuprofen, insulin, losartan, potassium chloride, sodium polystyrene sulfonate, spironolactone and valsartan) were monitored during a hyperkalemic event (serum potassium >5.5 mmol/L). Based on standard knowledge [18], the discontinuation pattern was expected to include ibuprofen, losartan, potassium chloride, spironolactone and valsartan; the intervention pattern was expected to include calcium gluconate, insulin, and sodium polystyrene sulfonate; and the maintenance pattern was expected to include amlodipine and hydrochlorothiazide. After all eligible hyperkalemic events were obtained from the database, all selected prescriptions (including the drug name in the Anatomical Therapeutic Chemical code, administration time, and prescriber) during the observation period were extracted. If baseline serum potassium (measured first after admission) was beyond the upper normal reference range, then the observation was excluded. Patients with chronic renal failure receiving dialysis were also excluded from the observations to prevent confounding by an underlying condition that could affect potassium homeostasis. The derived prescription patterns were compared to the standard knowledge described above.

#### 3.2 Agreement between prescriber knowledge and calculated prescription pattern

We could not be sure that all prescribers in the hospital were aware of the current clinical guidelines. Therefore, assessing the agreement between prescription patterns derived from the observations and the hyperkalemia management information in the literature may not be sufficient to confirm the hypothesized usefulness of prescription patterns. Therefore, we measured the degree of an individual prescriber's knowledge of hyperkalemia management and compared it with their prescription patterns. Three clinical case presentations were developed by three of the authors who are also physicians (a nephrologist, a pathologist, and a general physician; see questionnaire in **File S1**). A staff lecture on hyperkalemia management was announced to the internal medicine residents of the subject hospital. A quiz based on a questionnaire was conducted at the beginning of the lecture. The examinee residents were asked to modify the prescriptions of the aforementioned 10 drugs for each example hyperkalemic patient. The prescription patterns of the residents were calculated using their own prescription data for up to 1 year previously. Cohen's kappa was used to evaluate the degree of agreement between physician knowledge from the responses to the questionnaires with their prescription patterns. The minimum number of prescriptions of a drug for the agreement calculation was 10, because a prescription pattern derived from too few prescriptions would not be reliable.

#### 3.3 Validation study: application to CDAD

To determine whether prescription pattern could be used for clinical events other than hyperkalemia, we assessed CDAD, an important adverse event caused by prolonged use of broad-spectrum oral antibiotics. The standard care for CDAD is discontinuation of all antibiotics and administration of metronidazole or vancomycin [19]. Ordering the *C. difficile* toxin test suggests suspicion of CDAD. Therefore, we set both “ordering the *C. difficile* toxin test” and a “positive *C. difficile* toxin test” as clinical events. Four drugs, including two third-generation cephalosporins (cefotaxime and cefpiramide), clindamycin, and metronidazole, were monitored. Based on standard knowledge [19], cephalosporins and clindamycin were expected to show a discontinuation pattern, whereas an intervention pattern was expected for metronidazole. The PCI of each drug was calculated from the observations, and the prescription pattern was determined based on the PCI. The derived prescription patterns were compared to the standard knowledge described above.

### 4. Plots of prescription pattern according to degree of severity and clinical condition

The severity of an event usually lies in a spectrum, and guidelines vary according to event severity. We devised a method of visualizing the varying prescription patterns in a single graph according to degree of severity. The plot included PCI on the y-axis and event severity on the x-axis. Parameters were potassium level >4.50 mmol/L, and the 10 drugs in section ‘2.1 Hypothesis testing’ were plotted according to increasing serum potassium level (4.50 to 7.00 mmol/L in 0.25 mmol/L intervals).

The graph incorporated clinical condition on the x-axis for tests with a nominal result (*e*.*g*., “before ordering a toxin test”, “ordering a toxin test”, and “confirmation of toxin test result”). Observations identical to those in section ‘3.3. Validation study: application to CDAD’ were used.

### 5. Statistical analyses and software tools

Cohen's kappa was used to evaluate the degree of agreement between physician knowledge and prescription pattern. We used the Eclipse tools, version 3.7.1 (IBM, Riverton, NJ, USA) for JAVA programming and MS-SQL 2005 (Microsoft, Redmond, WA, USA) as the database management system. The R package (R Development Core Team, Vienna, Austria) was used for statistical analyses.

## Results

### 1. Study population

The demographic characteristics of the study participants are presented in **Table 1**. A total of 3868, 785, and 1785 observations (individual patients = 1698, 348, and 1288, respectively) were enrolled in each of the following studies in sequence.

**Table 1:** Characteristics of the study population.

	Study 1^a^	Study 2^b^	Study 3^c^
*n* d (observation count)	1,698 (3,868)	348 (785)	1,288 (1,785)
Age^e^, mean ± SD	39.58±29.81	46.43±29.17	52.73±22.32
Male, *n* d (%)	1,056 (62.19)	222 (63.79)	690 (53.57)
Underlying disease, *n* d ^,^ f (%)			
Renal failure	82 (4.83)	24 (6.90)	85 (6.60)
Heart failure	47 (2.77)	15 (4.31)	27 (2.10)
Diabetes	189 (11.13)	52 (14.94)	151 (11.72)

a Hypothesis testing: application to hyperkalemia.

b Agreement between prescriber knowledge and prescription pattern.

c Validation study: application to *Clostridium difficile*-associated diarrhea.

d Individual patients.

e Age at first admission of an individual patient.

f Underlying diseases were identified by the International Classification of Diseases 10^th^ Revision (ICD-10) codes: renal failure (I12.0, I13.0, I13.1, N17.x–19.x), heart failure (I11.0, I13.0, I13.2, I50.0, I50.1, I50.9) and diabetes (E10.x–E14.x).

SD, standard deviation.

### 2. Study 1: Hypothesis testing: application to hyperkalemia

**Figure 2:**
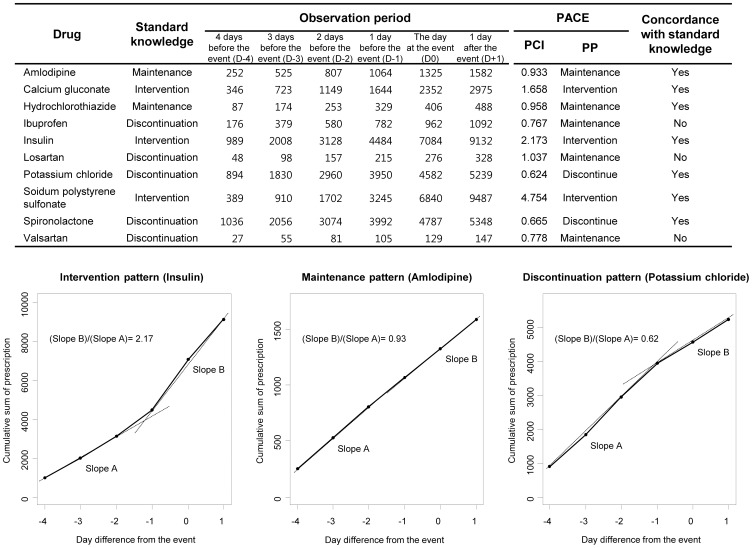
Prescription pattern for hyperkalemia measured by the PACE algorithm. Prescription patterns were categorized into three types based on the prescription change index (PCI): intervention pattern (PCI>1.500, bottom left), maintenance pattern (0.667<PCI≤1.500, bottom center), and discontinuation pattern (PCI≤0.667, bottom right). Prescribing of calcium gluconate, insulin, and sodium polystyrene sulfonate increased after the occurrence of an event. Potassium chloride and spironolactone showed the discontinuation pattern. Amlodipine, hydrochlorothiazide, ibuprofen, losartan, and valsartan were maintained regardless of event occurrence (maintenance pattern). D-4, D-2, and D-1 represent 4, 2, and 1 day(s) before the event, respectively; D0 represents the day on which the event occurred; D+1 represents 1 day after the event.

The first proof-of-concept study was designed to test whether the prescription patterns derived EHR data corresponded to current knowledge on hyperkalemia (serum potassium >5.5 mmol/L) management. In accordance with current practice guidelines on hyperkalemia [18], prescriptions for potassium chloride (PCI = 0.624) and spironolactone (PCI = 0.665) were discontinued after hyperkalemia occurred, whereas prescriptions for calcium gluconate (PCI = 1.658), insulin (PCI = 2.173), and sodium polystyrene sulfonate (PCI = 4.754) increased; prescriptions for amlodipine (PCI = 0.933) and hydrochlorothiazide (PCI = 0.958) were maintained as expected. However, ibuprofen (PCI = 0.767), losartan (PCI = 1.037), and valsartan (PCI = 0.778) were not discontinued as expected (**Figure 2** **and Figure S1 in File S1**).

### 3. Study 2: Agreement between prescriber knowledge and calculated prescription pattern

The second study was designed to determine whether the prescription pattern was an accurate representation of the hyperkalemia management knowledge of physicians rather than the standard hyperkalemia management knowledge. The enrolled internal medicine residents (*n* = 16) from the subject hospital were asked to modify the prescriptions of the 10 drugs mentioned above for three example patients with hyperkalemia (see questionnaire in **File S1**). All enrolled residents responded correctly with regard to use of amlodipine, hydrochlorothiazide, insulin, potassium chloride, and spironolactone for a hyperkalemia event (**Table 2**). However, the rate of correct responses regarding the use of ibuprofen, sodium polystyrene sulfonate, and valsartan was 0.875. The correct response rates regarding calcium gluconate and losartan were lowest, at 0.813. Agreement between prescriber knowledge of hyperkalemia management and that calculated by the PACE algorithm from EHR data of the preceding year was substantial, with a kappa of 0.714 (**Table S1 in File S1**) [20].

**Table 2:** Prescriber hyperkalemia knowledge regarding therapeutic decisions related to 10 hyperkalemia-related drugs, as determined by questionnaire.

Drug	Correct response rate
Amlodipine	1.000
Calcium gluconate	0.813
Hydrochlorothiazide	1.000
Ibuprofen	0.875
Insulin	1.000
Losartan	0.813
Potassium chloride	1.000
Sodium polystyrene sulfonate	0.875
Spironolactone	1.000
Valsartan	0.875

### 4. Study 4: Validation study: application to CDAD

**Figure 3:**
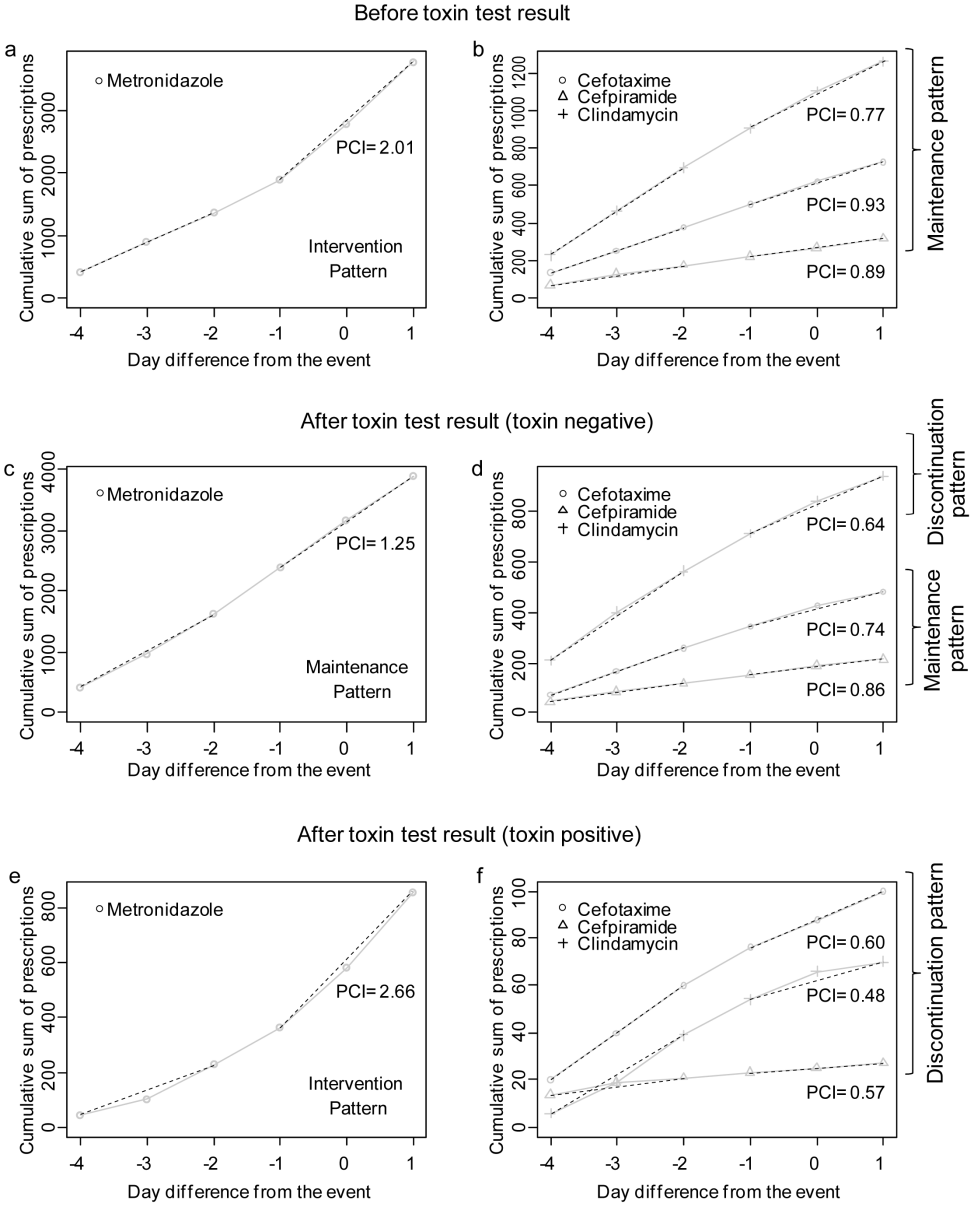
Changes in prescription patterns according to *Clostridium difficile*-associated diarrhea (CDAD). (a) and (b): Prescription patterns of metronidazole (a), cefotaxime, cefpiramide, and clindamycin (b) on the day on which a *C. difficile* toxin test was ordered (before the toxin test result). (c–f): prescription patterns on the day on which toxin test results were confirmed; (c) and (d): *C. difficile* toxin negative; (e) and (f): *C. difficile* toxin positive.

The third study was designed to determine whether the proposed method could be used for other clinical events; CDAD was used as an example. When index date was set to the date on which the *C. difficile* toxin test was ordered, use of metronidazole increased, whereas use of broad-spectrum antibiotics (cefotaxime, cefpiramide and clindamycin) was maintained (**Figure 3a and 3b**). After confirmation of the result of the *C. difficile* toxin test (average 3.24 days after ordering the test), the patterns of antibiotic use differed according to the test result. When the *C. difficile* toxin test was negative, use of cefotaxime, cefpiramide and metronidazole were maintained, whereas clindamycin was discontinued (**Figure 3c and 3d**). When the *C. difficile* toxin test was positive, use of metronidazole increased markedly (PCI = 2.66) but prescriptions for cefotaxime, cefpiramide, and clindamycin were discontinued (**Figure 3e and 3f**). These changes are in accordance with the standard CDAD guidelines [19].

### 5. Summary prescription pattern according to degree of severity or clinical condition

**Figure 4:**
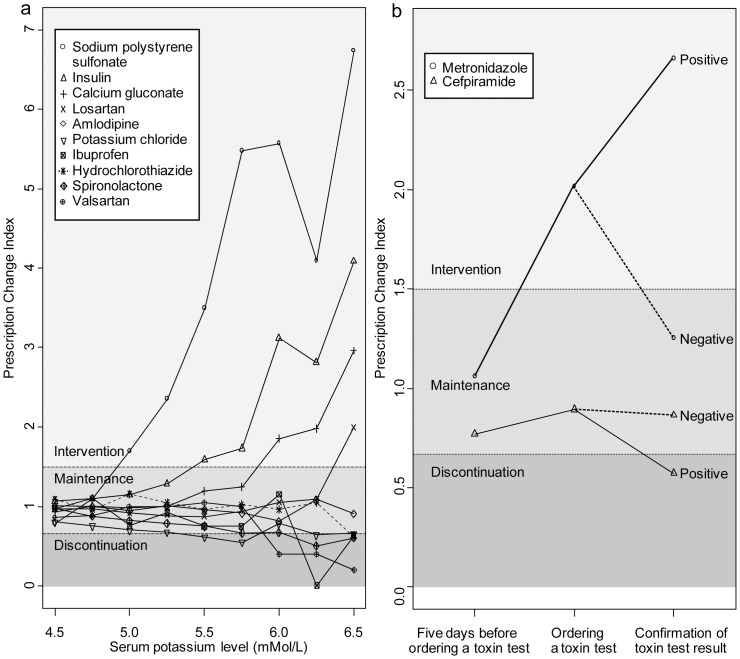
Graphs of prescription pattern according to event severity and clinical condition. (a): Changes in the prescription change index (PCI) according to serum potassium level. Prescriptions for sodium polystyrene sulfonate, insulin, and calcium gluconate increased with increasing serum potassium level. In contrast, use of ibuprofen, potassium chloride, spironolactone, and valsartan decreased. (b): Changes in PCI according to clinical condition. Metronidazole was used to treat *Clostridium difficile*-associated diarrhea (CDAD) beginning when the *C. difficile* toxin test was ordered by the prescriber. However, if the test was negative, use of metronidazole decreased. In contrast, if the result was positive, then cefpiramide prescription exhibited the discontinuation pattern.

Plotting of PCIs by serum potassium level provided a birds-eye view of prescription strategies at various potassium levels (**Figure 4a**). We observed a tendency to discontinue potassium chloride at 5.50 mmol/L and to discontinue spironolactone at 6.25 mmol/L. Sodium polystyrene sulfonate and insulin were used as therapeutic agents for hyperkalemia at a level of 5.00–5.50 mmol/L, and prescriptions of insulin increased rapidly beginning at a serum potassium level of 5.75–6.0 mmol/L. Calcium gluconate was used to lower a serum potassium level >6.00 mmol/L. Ibuprofen and valsartan were discontinued at 6.00–6.25 mmol/L serum potassium. Prescriptions for amlodipine, a negative control drug (unrelated to potassium metabolism), showed no change (maintenance pattern) according to potassium level. These findings are in agreement with the clinical guidelines for hyperkalemia. However, losartan and hydrochlorothiazide prescription practices were not in accordance with the relevant guidelines.

A summary graph of the *C. difficile* toxin test results was plotted (**Figure 4b**). When *C. difficile* infection was confirmed by a positive *C. difficile* toxin test result, metronidazole prescriptions showed the intervention pattern, whereas that for cefpiramide changed to the discontinuation pattern, as expected. In contrast, when *C. difficile* infection was not suspected (5 days before ordering a toxin test, or negative toxin test), neither the metronidazole intervention nor cefpiramide discontinuation patterns were observed, as expected.

## Discussion

We developed a novel algorithm that enables identification of the prescription patterns of prescribers relative to a specific clinical event. The observed prescription patterns are in accordance with current hyperkalemia knowledge. Moreover, the prescription patterns measured by our algorithm were well correlated with the hyperkalemia knowledge of the prescribing physicians. The CDAD prescription patterns are also in accordance with the relevant clinical guidelines.

The PACE algorithm was inspired by the earlier LEOPARD algorithm. Both compare accumulated prescription counts before and after an event. However, two differences between these algorithms exist. First, PACE monitors the prescription patterns of physicians using EHR data, whereas LEOPARD removes protopathic bias from adverse drug reaction (ADR) signals generated by other methods, such as the Longitudinal Gamma Poisson Shrinker or self-controlled case series. These ADR signal detection methods compare the rate of events during exposure to the rate when not exposed, and are therefore vulnerable to confounding by indication, because therapeutic drugs for a clinical event may be prescribed more frequently when the event occurs. The LEOPARD algorithm discriminates drugs that might cause an ADR from those that might be used for the treatment of an ADR by comparing the accumulated number of prescriptions before and after the event. A drug prescribed more frequently after the event than before is indicated by the algorithm to be used for treatment. Based on the LEOPARD algorithm, we hypothesized that prescriber intention for a given drug can be determined quantitatively from the EHR data by analyzing the slope of the ratio of its use before and after the relevant clinical event. The second major difference between the algorithms is the method of calculation of the decision point. The PACE algorithm measures the change in prescription number using the slope of the cumulative sum of prescriptions before and after an event (**Figure 1**). In contrast, LEOPARD uses a binomial test to evaluate the statistical significance of deviations from the expected distribution. Both methods have their strengths and weaknesses. The binomial test provides an unequivocal statistical basis for determination of prescription pattern; furthermore, the number of prescriptions involved or cases enrolled can be considered because the confidence interval can be determined, which is not possible using PACE. In contrast, the calculation involved in the PACE algorithm is simple in that it makes use of the PCI derived from the slope ratio, and can be applied to a typical EHR system with no need for use of statistical software or statistical knowledge. An intuitive birds-eye-view graph of changes in prescribing following laboratory tests can be plotted, and interpretation of PCI is more intuitive than that of *p* values. Furthermore, a semi-quantitative comparison of PCI values is possible; *e*.*g*., an intervention pattern with a PCI of 5.0 indicates a more intense intervention than that with a PCI of 1.6. Thus, we used the PCI rather than the binomial test to identify prescription pattern using the PACE algorithm. Results of both methods were similar as determined by *post hoc* analysis (see Supporting Information, **Table S2, S3, and S4 in File S1**).

Various quality indicators have been used to estimate the quality of healthcare: structure indicators (type and amount of resources), process indicators (assessment of what the provider did for the patient and how well it was done), and outcome indicators (health state or events following care and that may be affected by healthcare) [21]. Structure and outcome indicators assess the current state using rates of admission, mortality, or survival. Process indicators assess a series of healthcare activities. However, many process indicators used today are rate-based (*e*.*g*., the proportion of patients with myocardial infarction who received thrombolysis) [21]. Rate-based process indicators have limitations in terms of assessing whether the appropriate action was performed at the appropriate time. Therefore, many process indicators are sequential indicators that evaluate disease and event sequentially (*e*.*g*., “IF a vulnerable elder is prescribed an angiotensin-converting enzyme (ACE) inhibitor, THEN he or she should have serum creatinine and potassium monitored within 2 weeks after initiation of therapy and at least yearly thereafter” [22]). Although these sequential indicators can assess timely action of physicians, they may not guarantee that the responsible physicians have proper awareness of that action. The rate of potassium level monitoring after ACE inhibitor initiation can be high, if we assume that the physicians prefer to order routine laboratory tests regardless of the event, despite their lack of knowledge of the quality indicator or relationship between the ACE inhibitor and hyperkalemia. Furthermore, measuring such indicators usually requires a variety of data and resources and may involve manual labor. As every indicator requires its own data and decision structure based on established knowledge, there are limitations to be used as a global measurement tool. In contrast, the PACE algorithm is simple to use and interpret. It only requires prescription data from 4 days before to 2 days after a clinical event. Diverse clinical events or physicians' behaviors can be monitored using the PACE algorithm, because it is a data-driven knowledge discovery tool rather than a fixed indicator requiring specific data for a specific event. The algorithm provides information on the physicians' recognition of the clinical guidelines by capturing their intention to increase, decrease or maintain drug treatments for events at a specific point in time. Therefore, the PACE algorithm can potentially be used to monitor various prescription qualities.

Unlike existing indicators, the PACE algorithm has uniform decision criteria, and uses only prescription counts from the EHR data. This simplicity of the PACE algorithm facilitates automation of all component steps, including monitoring the PCI and prescription pattern in near-real time. It can be systematized and run using most present health information systems. The PCI summary graphs provide unprecedented insight into treatment strategy according to clinical event severity or clinical condition. Although we chose 10 drugs and their prescription–laboratory test pair, the algorithm can be used for any drug or any order–order pair (e.g., prescription–physical examination or procedure–pathology pair).

In a series of proof-of-concept studies, we evaluated the validity and usability of the algorithm. Prescription patterns for amlodipine, calcium gluconate, hydrochlorothiazide, insulin, potassium chloride, sodium polystyrene sulfonate and spironolactone were concordant with established hyperkalemia management knowledge. Although ibuprofen and valsartan did not show the expected discontinuation pattern for mild hyperkalemia, they changed to the expected discontinuation pattern after moderate hyperkalemia (**Figure 4a**). However, the losartan prescription pattern did not agree with standard knowledge and showed the opposite pattern (intervention pattern) to that expected for severe hyperkalemia (**Figure 4a**). In the second proof-of-concept study, we evaluated clinician knowledge and its agreement with the measured prescription patterns. The residents enrolled in the study were aware of whether they should discontinue, maintain, or increase use of most of the drugs upon occurrence of hyperkalemia. However, the correct response rates of losartan and calcium gluconate were lowest (0.813). This might cause the spurious losartan prescription pattern. In contrast, although calcium gluconate showed an equally low correct response rate, the calculated prescription pattern was consistent with standard knowledge. The failure to mention a change in electrocardiogram or cardiac symptoms in the questionnaire may have confused the residents, because these are required for the prescription of calcium gluconate. The fact that prescriptions by residents can be modified by their supervisor, regardless of their own knowledge of the event, may have decreased the correlation. Recall bias may have affected the results, because our findings are dependent on the responses of residents to questionnaires regarding hyperkalemia. Although the questionnaires regarding hyperkalemia were prepared carefully, the possibility of information bias cannot be excluded, because the clinical cases included in the questionnaire may have differed from actual observations in practice. The final proof-of-concept study indicated that the PACE algorithm could also be applied to CDAD. The prescription patterns in different clinical situations (suspicion of CDAD, negative or positive toxin test) were in agreement with current clinical knowledge.

Among the available laboratory tests, hyperkalemia and CDAD were selected for the proof-of-concept studies. Hyperkalemia is a common but potentially life-threatening electrolyte disorder [23]. Thus, serum potassium measurement is usually included in routine laboratory testing. CDAD is a representative hospital-acquired disease associated with significant morbidity and mortality that requires an immediate change of antibiotics [24], [25]. Testing for CDAD is quite different from that for hyperkalemia. Serum potassium is often routinely measured even without any suspicion of hyperkalemia. However, ordering a *C. difficile* toxin test usually requires supporting evidence (for example, prior long-term use of broad-spectrum antibiotics and episodes of diarrhea etc.). It also takes several days before the results are received. Thus, two quite different laboratory tests, the serum potassium and *C. difficile* toxin test, were selected for more comprehensive proof of studies.

This study had a number of limitations. Although 0.667 (1.000/1.500) and 1.500 were the PCI cut-off values determined by consensus among the authors and confirmed by sensitivity analysis (see Supporting Information and **Figure S2 in File S1**), these values lack a concrete statistical basis. However, as demonstrated in the *post hoc* test (File S1) determination of prescription patterns using a binomial statistical test showed similar results. Additionally, the study participants were limited to inpatients of a teaching hospital. Outpatients cannot be considered in the algorithm, because the interval between an event and a hospital visit by an outpatient is both irregular and longer than that of an inpatient. As the hospital stay duration for inpatients is relatively short, the algorithm is limited in that it monitors only 6-day changes and does not detect events requiring long-term observation. Thus, the algorithm basically focuses on immediate response of physicians to acute reactions. To account for outpatient and long-term changes, the observation period should be modified. However, whether performance of the algorithm is conserved after modification of the observation period remains to be validated; thus, further study is required. Increasing or decreasing the drug dose while maintaining the frequency of administration is a widely used prescribing method in practice. However, our method did not consider such dose adjustment, because this would require more complex data sources and calculation time, which might hinder adoption of the method. We chose hyperkalemia and CDAD for the proof-of-concept studies, rather than more popular clinical conditions such as hypertension or asthma, because the EHR system in the subject hospital did not include blood pressure data until 2010 and pulmonary function test results had not been digitalized. Instead, the results of laboratory tests were available in the EHR, and represented the most reliable and objective data therein. As present EHR systems replace most paperwork, and record a variety of clinical data digitally, we believe that our method can be applied to other clinical events. Although we demonstrated the usefulness of the proposed PACE algorithm in a multi-stage proof-of-concept study, the algorithm should in future be validated using data from a variety of clinical settings.

In conclusion, the proposed PACE algorithm extracted the prescription patterns of prescribers from EHR data based on a specific clinical event. The prescription patterns were well correlated with prescriber knowledge and standard knowledge of the event. Prescribing behaviors according to event severity were plotted in a simple graph, which provides an overview of prescription behavior. As the PACE algorithm is based on a simple equation and requires little EHR data, we believe it to be transferrable to other EHR systems. The PACE algorithm can thus be used to evaluate prescriber adherence to clinical guidelines and so will likely facilitate improvement in prescription quality.

## Supporting Information

File S1 **Supporting information document including Table S1, S2, S3, S4, Figure S1 and S2.**
(DOC)
