# Supplementary material for: A Quantitative Method for Assessment of Prescribing Patterns Using Electronic Health Records
Source: PLoS One. 2013 Oct 10;8(10):e75214. doi: 10.1371/journal.pone.0075214 (PMC3794932; doi:10.1371/journal.pone.0075214)
Supplement: File S1 — Supporting information document including Table S1, S2, S3, S4, Figure S1 and S2. (DOC) [file pone.0075214.s001.doc]

**SUPPORTING INFORMATION**

**A Quantitative Method for Assessment of Prescribing Patterns using Electronic Health Records**

Authors: D Yoon, I Park, MJ Schuemie, MY Park, JH Kim, and RW Park

**Table S1.** Agreement between prescriber hyperkalemia knowledge as measured by questionnaire and prescription pattern as determined by prescription change index.

|  | | Prescriber knowledge  (by questionnaire^a^) | | Agreement^c^  (Cohen’s kappa) |
| --- | --- | --- | --- | --- |
|  |  | Yes | No |  |
| Prescription pattern by prescription change index^b^ | Yes | 22 | 4 | 0.71 |
|  | No | 2 | 15 |  |
| ^a^Prescriber hyperkalemia knowledge was measured by 10 questions regarding therapeutic decisions for 10 related drugs.  ^b^Prescription patterns determined by prescription change index calculated from prescriber prescription data. | | | | |

**Figure S1.** Prescription pattern of 7 drugs for hyperkalemia measured by the PACE algorithm.


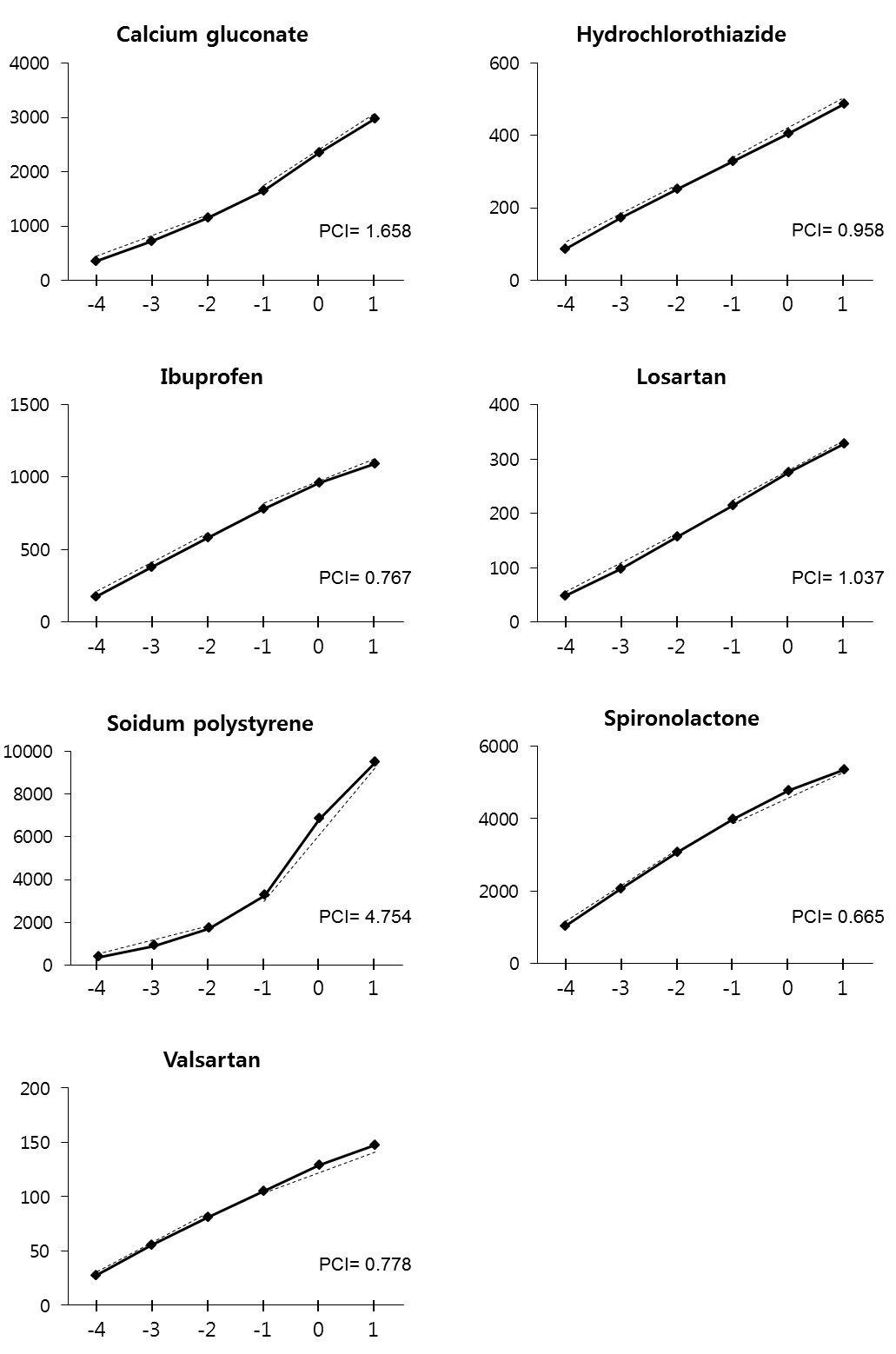


**Comparison of prescription pattern determination methods: prescription change index (PCI) *versus* binomial test**

We conducted a *post hoc* binomial test to determine prescription patterns. The data set and study design were identical to those used in the main analyses. We used a one-sided binomial test with the null hypothesis that the distribution of prescriptions over the two windows is correlated within the observation period. Because the observation period was 4 days (D-4–D-1) before the event and 2 days (D0–D+1) after the event, the distribution of prescriptions would be expected to be 4:2 if the event and prescribed drug were independent. Two one-sided binomial tests with an alternative hypothesis were used: the true probability of a prescription after the event is greater than the expected distribution of the intervention pattern; and the true probability of prescription after the event is less than the expected distribution of the discontinuation pattern.

The results using the PCI and that by the binomial test are presented in Table S2–S4. Table S2 shows the results of “hypothesis testing: application to hyperkalemia”, Tables S3 shows the “agreement between prescriber’s knowledge and prescription pattern”, and Table S4 shows the “validation study: application to CDAD.” Results of both the PACE algorithm and binomial test were similar. Nine of the 10 patterns were coincident with hypothesis testing (Table S2); thirty-six of 43 patterns were coincident with the agreement evaluation (Table S3); and nine of 12 patterns were coincident with the validation study.

| **Table S2.** Prescription patterns for hyperkalemia as determined by the prescription change index and binomial test. | | | | | | | | | | | | | | | | |
| --- | --- | --- | --- | --- | --- | --- | --- | --- | --- | --- | --- | --- | --- | --- | --- | --- |
| Drug | Standard knowledge  (expected pattern) | Cumulative sum of prescription count | | | | | |  | PACE | | |  | Binomial test | | |  |
|  |  | D-4 | D-3 | D-2 | D-1 | D0 | D+1 |  | PCI | Prescription  pattern | Concordance with standard knowledge |  | *p*^*^ (Intervention) | *p*^*^ (Discontinuation) | Prescription  pattern | Concordance with standard knowledge |
| Amlodipine | Maintenance | 252 | 525 | 807 | 1064 | 1325 | 1582 |  | 0.93 | Maintenance | Yes |  | 0.70 | 0.32 | Maintenance | Yes |
| Calcium gluconate | Intervention | 346 | 723 | 1149 | 1644 | 2352 | 2975 |  | 1.66 | Intervention | Yes |  | <0.01 | 1.00 | Intervention | Yes |
| Hydrochlorothiazide | Maintenance | 87 | 174 | 253 | 329 | 406 | 488 |  | 0.96 | Maintenance | Yes |  | 0.65 | 0.38 | Maintenance | Yes |
| Ibuprofen | Discontinuation | 176 | 379 | 580 | 782 | 962 | 1092 |  | 0.77 | Maintenance | No |  | 1.00 | <0.01 | Discontinuation | Yes |
| Insulin | Intervention | 989 | 2008 | 3128 | 4484 | 7084 | 9132 |  | 2.17 | Intervention | Yes |  | <0.01 | 1.00 | Intervention | Yes |
| Losartan | Discontinuation | 48 | 98 | 157 | 215 | 276 | 328 |  | 1.04 | Maintenance | No |  | 0.35 | 0.69 | Maintenance | No |
| Potassium chloride | Discontinuation | 894 | 1830 | 2960 | 3950 | 4582 | 5239 |  | 0.62 | Discontinuation | Yes |  | 1.00 | <0.01 | Discontinuation | Yes |
| Sodium polystyrene sulfonate | Intervention | 389 | 910 | 1702 | 3245 | 6840 | 9487 |  | 4.75 | Intervention | Yes |  | <0.01 | 1.00 | Intervention | Yes |
| Spironolactone | Discontinuation | 1036 | 2056 | 3074 | 3992 | 4787 | 5348 |  | 0.67 | Discontinuation | Yes |  | 1.00 | <0.01 | Discontinuation | Yes |
| Valsartan | Discontinuation | 27 | 55 | 81 | 105 | 129 | 147 |  | 0.78 | Maintenance | No |  | 0.91 | 0.13 | Maintenance | No |
| D-4, D-2, and D-1 represent 4, 2, and 1 day(s) before the event, respectively; D0 represents the day on which the event occurred; D+1 represents 1 day after the event; PCI, prescription change index; ^*^*p*-value by one-sided binomial test. | | | | | | | | | | | | | | | | |

| **Table S3.** Agreement between prescriber hyperkalemia knowledge (questionnaire) and prescription pattern (binomial test) for cases with the expected intervention pattern. | | | | |
| --- | --- | --- | --- | --- |
|  | | Prescriber knowledge  (by questionnaire^a^) | | Agreement  (Cohen’s kappa) |
|  |  | Yes | No |  |
| Prescription pattern  by binomial test^b^ | Yes | 20 | 3 | 0.67 |
|  | No | 4 | 16 |  |
| ^a^Prescriber knowledge of hyperkalemia was measured by means of 10 questions regarding therapeutic decisions related to 10 drugs.  ^b^Prescription patterns as determined by binomial test. | | | | |

| **Table S4.** Comparison of prescription patterns determined by prescription change index (PACE algorithm) with those by binomial test for *Clostridium difficile*-associated diarrhea. | | | | | | | | | | | | | | |  |
| --- | --- | --- | --- | --- | --- | --- | --- | --- | --- | --- | --- | --- | --- | --- | --- |
| Event | | Drug | Cumulative sum of prescription count | | | | | |  | PACE | |  | Binomial test | | |
|  |  |  | D-4 | D-3 | D-2 | D-1 | D0 | D+1 |  | PCI | Prescription  pattern |  | *p** (Intervention) | *p** (discontinuation) | Prescription  pattern |
| Ordering toxin test | | Cefotaxime | 135 | 251 | 377 | 499 | 619 | 724 |  | 0.93 | Maintenance |  | 0.91 | 0.11 | Maintenance |
|  |  | Cefpiramide | 71 | 130 | 175 | 223 | 266 | 316 |  | 0.89 | Maintenance |  | 0.94 | 0.08 | Maintenance |
|  |  | Clindamycin | 228 | 464 | 692 | 904 | 1101 | 1259 |  | 0.77 | Maintenance |  | 1.00 | <0.01 | Discontinuation |
|  |  | Metronidazole | 423 | 898 | 1364 | 1877 | 2780 | 3773 |  | 2.01 | Intervention |  | <0.01 | 1.00 | Intervention |
| Confirmation of *Clostridium* *difficile* toxin test result | Negative | Cefotaxime | 73 | 165 | 260 | 343 | 426 | 481 |  | 0.74 | Maintenance |  | 0.99 | 0.02 | Discontinuation |
|  |  | Cefpiramide | 43 | 84 | 116 | 151 | 186 | 214 |  | 0.86 | Maintenance |  | 0.90 | 0.13 | Maintenance |
|  |  | Clindamycin | 209 | 401 | 565 | 715 | 844 | 944 |  | 0.64 | Discontinuation |  | 1.00 | <0.01 | Discontinuation |
|  |  | Metronidazole | 419 | 980 | 1623 | 2391 | 3184 | 3897 |  | 1.25 | Maintenance |  | <0.01 | 1.00 | Intervention |
|  | Positive | Cefotaxime | 20 | 40 | 60 | 76 | 88 | 100 |  | 0.60 | Discontinuation |  | 0.98 | 0.03 | Discontinuation |
|  |  | Cefpiramide | 14 | 19 | 21 | 23 | 25 | 27 |  | 0.57 | Discontinuation |  | 0.99 | 0.03 | Discontinuation |
|  |  | Clindamycin | 6 | 19 | 39 | 54 | 66 | 70 |  | 0.48 | Discontinuation |  | 0.98 | 0.04 | Discontinuation |
|  |  | Metronidazole | 43 | 104 | 230 | 363 | 579 | 860 |  | 2.66 | Intervention |  | <0.01 | 1.00 | Intervention |
| D-4, D-2, and D-1 represent 4, 2, and 1 day(s) before the event, respectively; D0 represents the day on which the event occurred; D+1 represents 1 day after the event; PCI, prescription change index; **p*-value by one-sided binomial test. | | | | | | | | | | | | | | | |

**Sensitivity analysis**

We conducted sensitivity analyses of each of the first two proof-of-concept studies, “Hypothesis testing: application to hyperkalemia” and “Agreement between prescriber knowledge and prescription pattern”, by altering the cut-off values: x-fold reduction or increase in the sum of prescriptions before and after a clinical event from 1.25 to 2.00-fold in 0.25-fold intervals. Thus the cut-off values were altered from 1.25 to 2.00 to determine the intervention pattern, whereas for the discontinuation pattern they were altered from 0.80 to 0.50 (1.00/1.25, 1.00/1.50, 1.00/1.75, 1.00/2.00). The accuracy of the comparison of prescription pattern from EHR data and standard hyperkalemia knowledge was measured by varying the cut-off value.

Accuracy decreased from 0.90 to 0.40 with increasing cut-off values, whereas agreement between the prescription pattern and standard knowledge of hyperkalemia increased from 0.52 to 0.63 (Cohen’s kappa). Kappa values were identical at cut-offs of 1.50- or 1.75-fold; however, accuracy was greater at a cut-off of 1.50-fold.

**Figure S2.** Accuracy and correlation according to cut-off value. A “1.5-fold” cut-off provides a balance between accuracy and the correlation between the prescription pattern and standard hyperkalemia knowledge (Cohen’s kappa).


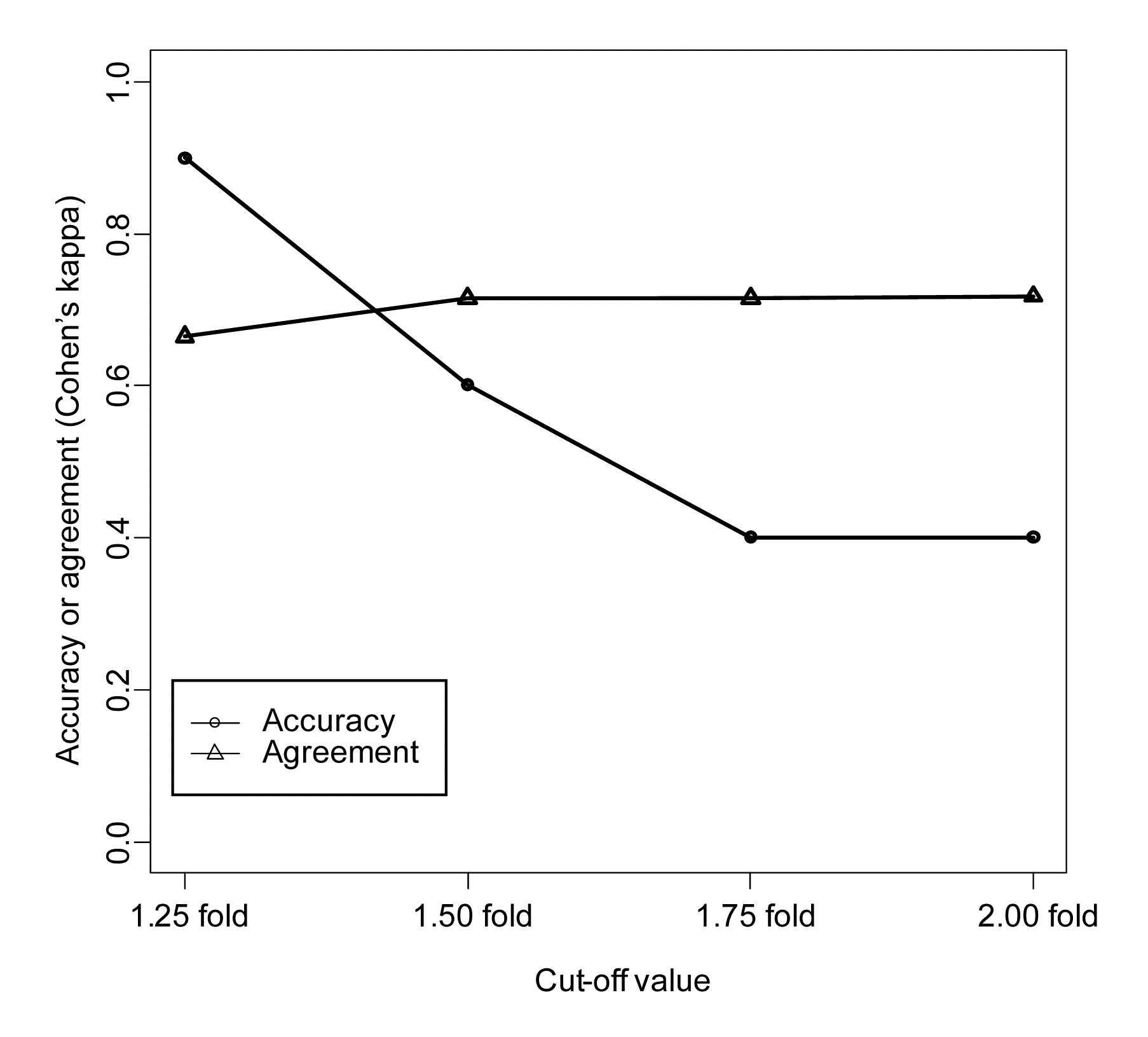


**Questions to evaluate prescriber knowledge of the therapeutic management of hyperkalemia**

Respond “Yes” or “No” after reading the following passages and questions.

1–4. The serum potassium level of a 56-year-old woman hospitalized for DM foot was 6.5 mMol/L. Her serum potassium level was 5.0 mMol/L 5 days ago. Blood pressure was normal and there was no edema or dehydration. She had taken the following medications until 3 days ago.

Losartan, Norvasc, Aldactone, Dichlozid

1. Discontinuation of Losartan (Yes/No)

2. Discontinuation of Norvasc (Yes/No)

3. Discontinuation of Aldactone (Yes/No)

4. Discontinuation of Dichlozid (Yes/No)

5–8. A 70-year-old male patient is receiving IV vancomycin for treatment of a MRSA infection. His serum creatinine level increased from 1.1 mg/dl 1 week ago to 3.0 mg/dl presently. His serum potassium level also increased from 5.2 mMol/L to 7.2 mMol/L. He had taken the following medications until 3 days ago.

Ketorolac tomethamin, ibuprofen, ramipril, insulin

5. Discontinuation of insulin (Yes/No)

6. Discontinuation of ibuprofen (Yes/No)

7. Administration of kayexalate (Kalimate^ⓡ^) (Yes/No)

8. Administration of calcium gluconate (Yes/No)

9–10. A 60 year-old male patient visited the Outpatient Department for hypertension. His serum potassium level was 7.3 mMol/L. He was taking the following medications.

K-contin, Valsartan, Lasix

9. Discontinuation of K-contin (Yes/No)

10. Discontinuation of Valsartan (Yes/No)
